# A PERMA-nent solution to understanding psychological wellbeing? Exploring the utility of the PERMA model in a university workplace

**DOI:** 10.3389/fpsyg.2025.1598910

**Published:** 2025-07-23

**Authors:** Xiao Hui Ng, Jeanie Chu, Kinjal Doshi

**Affiliations:** ^1^Department of Health and Wellbeing, National University of Singapore, Singapore, Singapore; ^2^INSiGHT Therapeutics and Consulting, Singapore, Singapore; ^3^Department of Psychology, National University of Singapore, Singapore, Singapore

**Keywords:** workplace wellbeing, mental health, PERMA, subjective quality of life, multicultural population

## Abstract

**Introduction:**

A diagnosis of a psychological health concern is associated with lower wellbeing and subjective quality of life (sQoL). However, there is limited literature examining whether individuals who suspect they have a psychological health condition (SPHC) experience similar challenges. The primary aim of this study is to investigate the relationship between SPHC, workplace wellbeing, and sQoL, with the goal of informing future workplace wellbeing interventions within a diverse university setting. A secondary aim is to explore whether additional aspects of wellbeing are valued by a multicultural population beyond the five core components defined in Martin Seligman’s Wellbeing Theory: positive emotion, engagement, relationships, meaning, and achievement.

**Methods:**

A 57-item survey was sent to a randomly generated list of 2,000 university staff to request for their participation. It gathered information on their understanding of wellbeing, presence of suspected psychological health conditions, level of distress, workplace psychological wellbeing, and quality of life.

**Results:**

First, individuals with SPHCs reported significantly lower levels of wellbeing and sQoL compared to those who did not suspect having a PHC. Second, workplace wellbeing was found to mediate the relationship between psychological distress and sQoL. Finally, qualitative analysis revealed five additional lay conceptualizations of wellbeing in addition to the five facets identified in PERMA, namely psychological health, physical health, balance, meeting basic needs, and autonomy.

**Discussion:**

That wellbeing mediates the relationship between psychological distress and sQoL suggests that workplace wellbeing interventions may be particularly important in improving sQoL, especially in individuals who experience SPHCs given that they also experience lower sQoL. That five other lay conceptualisations of wellbeing also emerged from the findings suggests that laypeople’s understanding of wellbeing adds a unique cultural and situational lens to the current understanding of this construct. Further implications are discussed.

## Introduction

Many studies focus on individuals with diagnosed psychological health concerns (PHCs), examining their prevalence and treatment. However, few studies investigate individuals who suspect they have psychological health conditions (SPHCs) but remain undiagnosed. This area warrants further research, as common PHCs such as depression (Faisal-Cury et al., 2022) and anxiety (Kasper, 2006) are often underdiagnosed or misdiagnosed and, consequently, undertreated, an issue with serious implications. Individuals with SPHCs are more likely to score above the clinical threshold for depression, anxiety, and post-traumatic stress, and to report poorer health-related quality of life and sleep quality compared to those who do not suspect having a PHC (Liu et al., 2020). Furthermore, the treatment gap for SPHC stands at 47% (Islam et al., 2022), which is concerning as longer treatment delays are associated with higher rates of comorbidity of other health conditions and increased mortality (McLaughlin, 2004).

However, beyond *clinical recovery* or symptomatic alleviation, it is also important to consider *personal recovery* or the striving toward increased psychological wellbeing (Slade, 2010), especially in individuals who may not have access to clinical services as they are not diagnosed with a psychological condition. While not specific to individuals who have SPHC, Vaingankar et al. (2020) demonstrated that while individuals with diagnosed PHCs generally indicate lower quality of life and poorer health outcomes as compared to individuals without diagnosed PHCs, having increased psychological wellbeing reduces the impact that diagnosed PHCs might have on these areas. This evolving understanding of recovery and wellbeing then raises the question of whether psychological health interventions can be delivered in environments other than traditional settings, such as clinics and hospitals, and by non-clinically trained individuals, if only an understanding of wellbeing can also be reached.

Wellbeing is understood as the experiencing of optimal psychological functioning (Deci and Ryan, 2006), with many attempts to define such positive functioning with a specific value framework (Diener, 1984), one of these being Martin Seligman’s Wellbeing Theory (WBT).

WBT posits that complete wellbeing, or *flourishing*, consists of five distinct factors, or PERMA, namely:

1. Positive emotion: Experiencing emotions such as hopefulness, happiness, and a sense of peace.
2. Engagement: Having meaningful work that keeps one purposefully occupied.
3. Relationships: Having warm, trusting relationships with others.
4. Meaning: Having a greater goal and sense of purpose in life.
5. Accomplishment: Experiencing a sense of accomplishment (Seligman, 2011).

While the PERMA factors constitute a broader concept of wellbeing, they also serve as distinct paths of striving toward a sense of wellbeing for different individuals (Seligman, 2011). Furthermore, as adults spend a significant portion of their time at work, workplace wellbeing is crucial. Effective workplace wellbeing interventions can reduce psychological distress and offer benefits like improved physical health, job satisfaction, and organizational commitment (Meyers et al., 2012; Kern et al., 2014). However, there are few empirically evaluated wellbeing interventions specifically designed for workplaces (Neumeier et al., 2017). Understanding how employees define and prioritize wellbeing is essential for crafting suitable interventions.

Regarding Quality of Life (QoL), while objective QoL focuses on economic indicators such as wealth and access to healthcare, subjective QoL (sQoL) looks at how an individual assesses their own QoL by their own standards (Cummins, 2000). Furthermore, subjective quality of life (QoL) appears to be more strongly associated with the intensity of distress experienced in relation to a health condition (Andenæs et al., 2004). While the individual concepts of wellbeing and QoL have been well-studied, their relationship is less understood. Research showed that both wellbeing and sQoL significantly contribute to overall QoL (Skevington and Böhnke, 2018). Skevington and Böhnke (2018) proposed an integrated model combining subjective wellbeing and sQoL, incorporating elements like financial resources, physical environment, emotions, and spirituality, which better explains QoL than either factor alone.

### Aims

This study first aims to add to the literature on SPHC and workplace wellbeing (WW) in a university work environment by investigating the relationship between SPHCs, WW, and sQoL. Secondly, it aims to explore whether there exist other aspects of wellbeing pertinent to a multicultural workplace that have not yet been identified by current research. Achieving these aims may identify areas of focus for WW interventions to aid in personal recovery beyond symptomatic alleviation, benefiting both individuals and workplaces.

### Hypotheses

Based on the aims, the following hypotheses were developed:

*H1*: Individuals with SPHCs will report lower WW compared to individuals without SPHCs (nSPHC).

*H2*: Individuals with SPHCs will report lower sQoL compared to individuals who do not experience SPHCs.

*H3*: WW will mediate the relationship between levels of distress (depression, anxiety, and stress) and sQoL.

To address the final aim, a qualitative component will explore how individuals in a multicultural workplace define wellbeing.

## Materials and methods

### Procedure

Invitations to respond to an online survey administered using Qualtrics were sent via e-mail to a randomly generated list of 2000 university staff stratified by department. A reminder email was sent 2 weeks after the first email to increase response rates.

### Measures

A 57-item questionnaire gathered data on six areas: basic demographic information, understanding of wellbeing, presence of suspected psychological health conditions, level of distress, workplace psychological wellbeing, and quality of life.

### Psychological health status

A question on suspected psychological health status was posed to participants. Participants could indicate that they suspected having a psychological health condition (SPHC), had no suspicions of PHC (nSPHC), or preferred not to say (SPNTS).

### Understanding of wellbeing

To understand participants’ subjective understanding of wellbeing, an open-ended question was included in the questionnaire asking, *“What does wellbeing mean for you personally?”*

### Subjective quality of life

The single-item Global QoL Scale by Hyland and Sodergren (1996) was included to measure participants’ sQoL. Participants were asked to move a slider from a scale of 0–100 to a point that best describes their QoL. The measure has demonstrated good test–retest reliability (Hyland and Sodergren, 1996).

### Depression, anxiety, and stress

The Depression-Anxiety-Stress Scale 21 was used to measure participants’ levels of depression, anxiety, and stress. Each item is scored on a four-point Likert scale. The 21-item self-report measure has three sub-scales designed to measure states of depression, anxiety, and stress and has been found to have strong internal consistency (*α* range = 0.86–0.96), convergent validity, and discriminant validity in large clinical samples (Brown et al., 1997). Strong internal consistency was observed for all scales (*α* range = 0.80–0.91), and discriminant validity was noted among various disorders in non-clinical samples (Sinclair et al., 2011).

### Workplace wellbeing

The 23-item Workplace PERMA profiler was used to measure subjective wellbeing in a workplace context. The scale was based on Seligman’s WBT (Butler and Kern, 2016) and has five key subscales: Positive Emotion, Engagement, Relationships, Meaning, and Accomplishment. Each subscale has three items scored on a 10-point scale. The sum of the items from the five primary subscales generates an overall workplace wellbeing score. Several studies have validated the profiler, demonstrating strong internal consistency except for the Engagement subscale (*α* = 0.66), as well as strong convergent and divergent validity (Ryan et al., 2019).

### Method of qualitative analysis

Codebook thematic analysis, specifically the Framework Method developed by Ritchie and Spencer (1994), was the selected form of analysis for the qualitative component. The Framework method’s flexibility in integrating both deductive and inductive thematic analysis approaches (Gale et al., 2013) made it an ideal means of analysis in the current study. The current research interest in the PERMA framework makes a deductive approach ideal, as themes can be drawn from the PERMA framework. An inductive approach also allowed for the emergence of new themes beyond the PERMA framework through open coding and refinement.

## Results

### Demographic information

A total of 214 responses were submitted, with 16 incomplete responses. The 198 completed responses were utilized for quantitative analysis (Table 1: Sociodemographic characteristics of population). Means and standard deviations for utilized measures across sociodemographic characteristics are reported in Table 2, and correlations for utilized measures are reported in Table 3.

**Table 1:** Sociodemographic characteristics of population.

Characteristic	Suspect has a psychological health condition		Do not suspect having a psychological health condition		Prefer not to say		Full sample	
	*n*	*%*	*n*	*%*	*n*	*%*	*n*	*%*
Age								
21–29 years old	10	5.1%	17	8.6%	3	1.5%	30	15.2%
30–39 years old	9	4.5%	54	27.3%	9	4.5%	72	36.4%
40–49 years old	7	3.5%	32	16.2%	4	2.0%	43	21.7%
50–59 years old	2	1.0%	36	18.2%	4	2.0%	42	21.2%
60–69 years old	0	0.0%	11	5.6%	0	0.0%	11	5.6%
Ethnicity								
Chinese	18	9.1%	102	51.5%	14	7.1%	134	67.7%
Malay	3	1.5%	16	8.1%	1	0.5%	20	10.1%
Indian	0	0.0%	12	6.1%	3	1.5%	15	7.6%
Others	7	3.5%	20	10.1%	2	1.0%	29	14.6%
Gender								
Male	9	4.5%	71	35.9%	4	2.0%	84	42.4%
Female	18	9.1%	77	38.9%	16	8.1%	111	56.1%
Other	1	0.5%	0	0.0%	0	0.0%	1	0.5%
Prefer not to say	0	0.0%	2	1.0%	0	0.0%	2	1.0%

**Table 2:** Means and standard deviations of measures across sociodemographic characteristics.

Characteristic	Subjective QoL		Depression (DASS-21)		Anxiety (DASS-21)		Stress (DASS-21)		Positive emotion (PERMA)		Engagement (PERMA)		Relationships (PERMA)		Meaning (PERMA)		Achievement (PERMA)		Total PERMA score	
	*M*	SD	*M*	SD	*M*	SD	*M*	SD	*M*	SD	*M*	SD	*M*	SD	*M*	SD	*M*	SD	*M*	SD
Age																				
21–29 years old	64.60	17.58	5.87	4.35	5.23	3.87	7.40	3.31	6.02	1.96	5.96	1.93	7.09	1.68	6.88	1.63	7.03	1.26	105.33	22.21
30–39 years old	66.68	16.21	5.26	4.17	4.08	3.03	6.60	3.83	6.24	2.14	6.55	1.63	6.68	1.86	6.72	1.97	7.19	1.40	106.65	23.20
40–49 years old	71.60	13.87	4.56	3.61	3.70	2.70	6.79	3.21	6.47	1.80	6.90	1.65	6.33	2.01	6.92	1.82	7.43	1.46	108.79	23.17
50–59 years old	71.95	14.45	3.90	3.89	3.10	2.63	5.24	2.78	6.65	2.20	7.44	1.71	6.60	2.11	7.37	1.87	7.81	1.27	114.45	24.34
60–69 years old	77.64	7.10	1.90	1.51	2.36	1.91	4.45	2.58	7.27	2.67	7.06	2.01	7.06	2.37	7.97	2.41	8.24	1.23	120.55	29.68
Ethnicity																				
Chinese	69.02	15.04	4.66	4.04	3.76	2.89	6.09	3.37	6.38	2.07	6.59	1.76	6.74	1.82	6.5	1.93	7.42	1.37	108.99	23.11
Malay	72.80	15.70	4.30	4.75	5.15	3.95	6.70	3.54	7.04	1.77	7.17	1.06	7.30	1.90	7.44	1.41	7.52	1.18	115.78	20.91
Indian	71.33	15.62	3.07	2.76	3.20	2.73	5.47	2.80	7.36	1.79	7.56	1.70	7.00	1.81	7.62	1.80	7.78	1.30	118.93	23.96
Others	66.17	17.32	6.17	3.49	3.83	3.09	7.79	3.64	5.28	2.25	6.83	2.10	5.77	2.41	6.62	2.05	7.12	1.59	102.04	27.16
Gender																				
Male	68.86	16.95	4.52	4.12	3.26	2.73	6.02	3.65	6.43	2.22	6.69	1.70	6.78	1.92	7.06	1.99	7.47	1.38	110.24	24.09
Female	69.46	14.41	4.84	3.94	4.30	3.21	6.53	3.26	6.37	2.01	6.60	1.82	6.73	2.01	6.93	1.86	7.35	1.39	108.56	23.98
Other	50.00	0.00	10.00	0.00	8.00	0.00	11	0.00	5.67	0.00	4.67	0.00	7.33	0.00	6.67	0.00	8.33	0.00	104.00	0.00
Prefer not to say	75.00	7.07	4.50	2.12	3.50	2.12	8.00	1.41	7.33	0.47	7.50	0.72	7.00	0.00	8.33	0.47	7.83	0.24	120.50	3.54

**Table 3:** Correlations between QoL score, DASS-21 scores, and PERMA scores.

Variable	*M*	SD	1	2	3	4	5	6	7	8	9	10
1. Subjective QoL	69.16	15.48	-									
2. Depression (DASS-21)	4.73	4.00	−0.58**	-								
3. Anxiety (DASS-21)	3.87	3.04	−0.21**	0.47**	-							
4. Stress (DASS-21)	6.35	3.43	−0.45*	0.67**	0.53**	-						
5. Positive emotion (PERMA)	6.40	2.07	0.61**	−0.69**	−0.23**	−0.47**	-					
6. Engagement (PERMA)	6.76	1.77	0.34**	−0.35**	−0.14**	−0.15**	0.61**	-				
7. Relationships (PERMA)	6.67	1.95	0.43**	−0.61**	−0.24**	−0.39**	0.77**	0.39**	-			
8. Meaning (PERMA)	7.00	1.90	0.39**	−0.55*	−0.23**	−0.32**	0.79**	0.69**	0.70**	-		
9. Achievement (PERMA)	7.41	1.38	0.31**	−0.41**	−0.23**	−0.30**	0.62**	0.63**	0.48**	0.67**	-	
10. Total PERMA score	109.34	23.82	0.53**	−0.64**	−0.27**	−0.41**	0.92**	0.77**	0.81**	0.91**	0.78**	-

**p < 0.01.

#### Suspected psychological health conditions

A total of 14.1% of participants reported a suspected PHC (SPHC *N* = 28), 75.8% had no SPHC (nSPHC *N* = 150), and 10.1% preferred not to say (SPNTS *N* = 20).

### Suspected psychological health condition and workplace wellbeing

A one-way ANOVA was performed to compare the effect of SPHC on workplace PERMA wellbeing scores with follow-up *post-hoc* tests using Tukey’s HSD Test for Multiple Comparisons.

#### Suspected psychological health conditions and wellbeing

There was a significant difference in overall PERMA wellbeing score [*F* (2, 192) = 10.70, *p* < 0.01] between the SPHC (*M* = 3.97, SD = 2.04) and nSPHC (*M* = 5.71, SD = 2.05) groups, *p* > 0.01, 95% CI = −32.39, −10.29. These results support H1 that individuals with SPHCs will report lower workplace wellbeing as compared to individuals without SPHCs.

Significant differences were also observed between the SPHC and nSPHC groups at all PERMA sub-facet levels. The nSPHCs group reported higher Positive Emotion, Engagement, Relationships, Meaning, and Achievement than the SPHC group (Table 4: One-way ANOVA; Table 5: *Post-hoc* tests).

**Table 4:** One-way ANOVA comparing the presence of suspected psychological health status on workplace wellbeing.

PERMA facet	SPHC		nSPHC		SPNTS		*F* (2, 192)	*p*	*η*2
	M	SD	M	SD	M	SD			
Positive emotion*	4.79	2.01	6.79	1.96	5.81	1.95	13.14	0.00*	0.12
Engagement*	5.85	2.04	6.91	1.70	6.93	1.53	4.51	0.01*	0.05
Relationships*	5.81	2.05	6.92	1.85	5.98	2.13	5.36	0.01*	0.05
Meaning*	5.86	1.66	7.24	1.88	6.83	1.88	6.63	0.00*	0.07
Achievement*	6.50	1.51	7.56	1.33	7.61	1.08	7.65	0.00*	0.07
Total PERMA score*	91.86	24.41	113.20	22.63	105.53	20.61	10.70	0.00*	0.10

*p < 0.05.

**Table 5:** *Post hoc* analysis of suspected psychological health status on workplace wellbeing.

Comparison groups									
PERMA facet	SPHC vs nSPHC			SPHC vs SPNTS			nSPHC vsSPNTS		
	Std Error	*p*	95% CI	Std Error	*p*	95% CI	Std Error	*p*	95% CI
Positive emotion	0.41	0.00*	−2.60, −1.04	0.59	0.19	−2.40, 0.36	0.48	0.11	−0.15, 2.11
Engagement	0.36	0.01*	−1.90, −0.22	0.52	0.09	−2.30, 0.13	0.42	1.00	−1.02, 0.97
Relationships	0.39	0.01*	−2.04, −0.18	0.57	0.95	−1.51, 1.17	0.47	0.11	−0.16, 2.04
Meaning	0.38	0.00*	−2.28, −0.48	0.55	0.19	−2.27, 0.33	0.45	0.63	−0.65, 1.48
Achievement	0.28	0.00*	−1.71, −0.41	0.40	0.02*	−2.05, −1.77	0.33	0.98	−0.82, 0.71
Total PERMA score	4.68	0.00*	−32.39, −10.29	6.75	0.11	−29.61, 2.27	5.53	0.35	−5.40, 20.74

*p < 0.05.

While no difference in overall PERMA scores was noted between SPHC and SPNTS groups, differences at the facet level were noted for achievement [*F* (2, 192) = 7.65, *p* < 0.01; SPHC (*M* = 6.50, SD = 1.51) and SPNTS (*M* = 7.61, SD = 1.08), *p* = 0.02, 95% CI = −2.05, −1.77], with the SPNTS group scoring higher than the SPHC group.

No difference in overall PERMA scores was noted between the nSPHC and SPNTS groups [*F* (2, 192) = 7.52, *p* = 0.35, 95% CI = −5.40, 20.74].

### Suspected psychological health conditions and sQoL

A one-way ANOVA was performed to compare the effect of SPHC on sQoL with follow-up *post-hoc* tests using Tukey’s HSD Test for Multiple Comparisons.

#### Suspected psychological health conditions and sQoL

Significant differences were noted in sQoL scores between at least two groups [*F* (2, 195) = 9.90, *p* > 0.01], with significant differences between the SPHC (*M* = 60.11, SD = 15.58) and nSPHC (*M* = 71.80, SD = 14.66) groups, *p* > 0.01, 95% CI = −18.90, −4.49, with the nSPHC group demonstrating a higher sQoL score than the SPHC group. These results supported H2, which states that individuals with SPHCs will report lower sQoL compared to individuals without SPHCs (Table 6: One-way ANOVA; Table 7: *Post-hoc* tests).

**Table 6:** One-way ANOVA comparing suspected psychological health status effect on sQoL.

Measure	SPHC		nSPHC		SPNTS		*F* (2, 195)	*p*	*η*2
	*M*	SD	*M*	SD	*M*	SD			
Quality of Life*	60.11	15.58	71.80	14.66	62.05	15.00	9.90	0.00	0.092

*p < 0.05.

**Table 7:** *Post hoc* tests for suspected psychological health status effect on sQoL.

Comparison groups									
Measure	SPHC vs nSPHC			SPHC vs SPNTS			nSPHC vs SPNTS		
	Std Error	*p*	95% CI	Std Error	*p*	95% CI	Std Error	*p*	95% CI
QoL	3.05	0.00*	−18.90, −4.49	4.34	0.90	−12.19, 8.31	3.53	0.02*	1.42, 18.08

*p < 0.05.

There was also a significant difference between the nSPHC and SPNTS groups (*M* = 62.05, SD = 15.00), *p* = 0.02, 95% CI = 1.42, 18.08, with SPNTS indicating lower sQoL.

There was no significant difference between the SPHC and SPNTS groups.

### Depression, anxiety, and stress levels, workplace wellbeing, and sQoL

SPSS add-on PROCESS Model 4 (Hayes, 2013) was run to assess whether workplace wellbeing PERMA scores mediate the relationship between self-reported depression, anxiety, and stress levels and sQoL.

#### Mediating effect of workplace wellbeing on the relationship between depression levels and sQoL

There was a significant direct effect of depression level, as measured with the DASS-21, on overall workplace wellbeing, *b* = −3.81, *t* = −11.64, *p* < 0.01 (95% CI = −4.45, −3.16). There was a further direct effect of overall workplace wellbeing on sQoL, *b* = 0.18, *t* = 3.62, *p* < 0.01, (95% CI = 0.08, 0.27), and a direct effect of depression score on sQoL, *b* = − 1.57, *t* = −5.48, *p* < 0.01, (95% CI = −2.14, −1.01).

**Figure 1:**
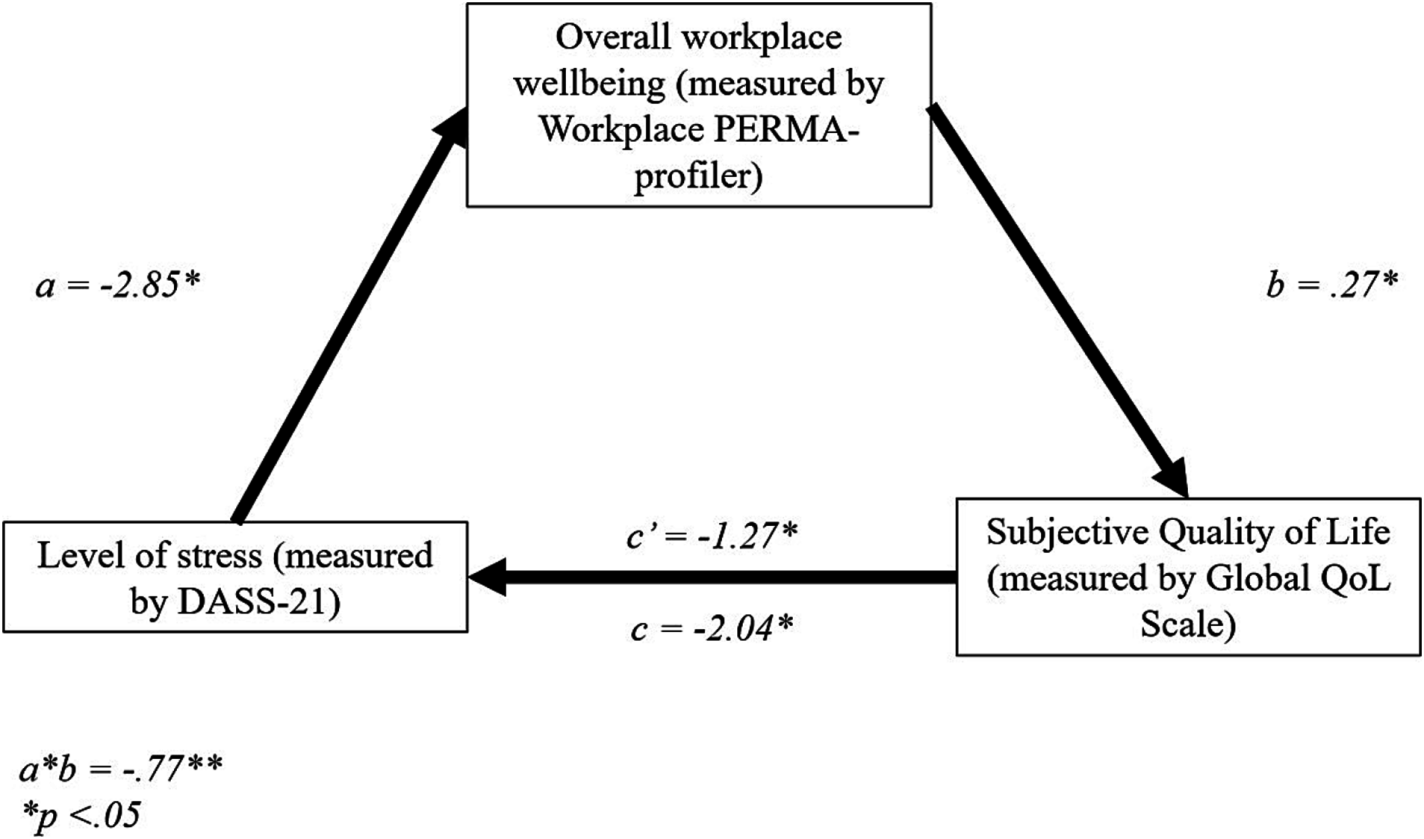
Stanzardised regression coefficients for the relationship between depression and sQoL. *p < 0.05, **p < 0.01.

The total effect model was significant, *b* = −2.24, *t* = −9.87, *p* < 0.01, 95% CI = −2.69, − 1.79. Finally, the indirect effect, *b* = −0.67, tested using a bootstrapping procedure with 5,000 samples (95% CI = −1.22, −0.22), was statistically significant (Figure 1). These results suggest that PERMA partially mediates the relationship between depression level and sQoL, thus supporting H_3_.

#### Mediating effect of workplace wellbeing on the relationship between anxiety levels and sQoL

There was a significant direct effect of anxiety level, as measured with the DASS-21, on overall workplace wellbeing, *b* = −2.11, *t* = −3.87, *p* < 0.01 (95% CI = −3.18, −1.03). There was a further direct effect of overall workplace wellbeing on sQoL, *b* = 0.33, *t* = 0.04, *p* < 0.01, (95% CI = 0.25, 0.41). However, the direct effect of anxiety level on sQoL was not significant, *b* = −0.42, *t* = −1.31, *p* = 0.19, (95% CI = −1.06, 0.22).

**Figure 2:**
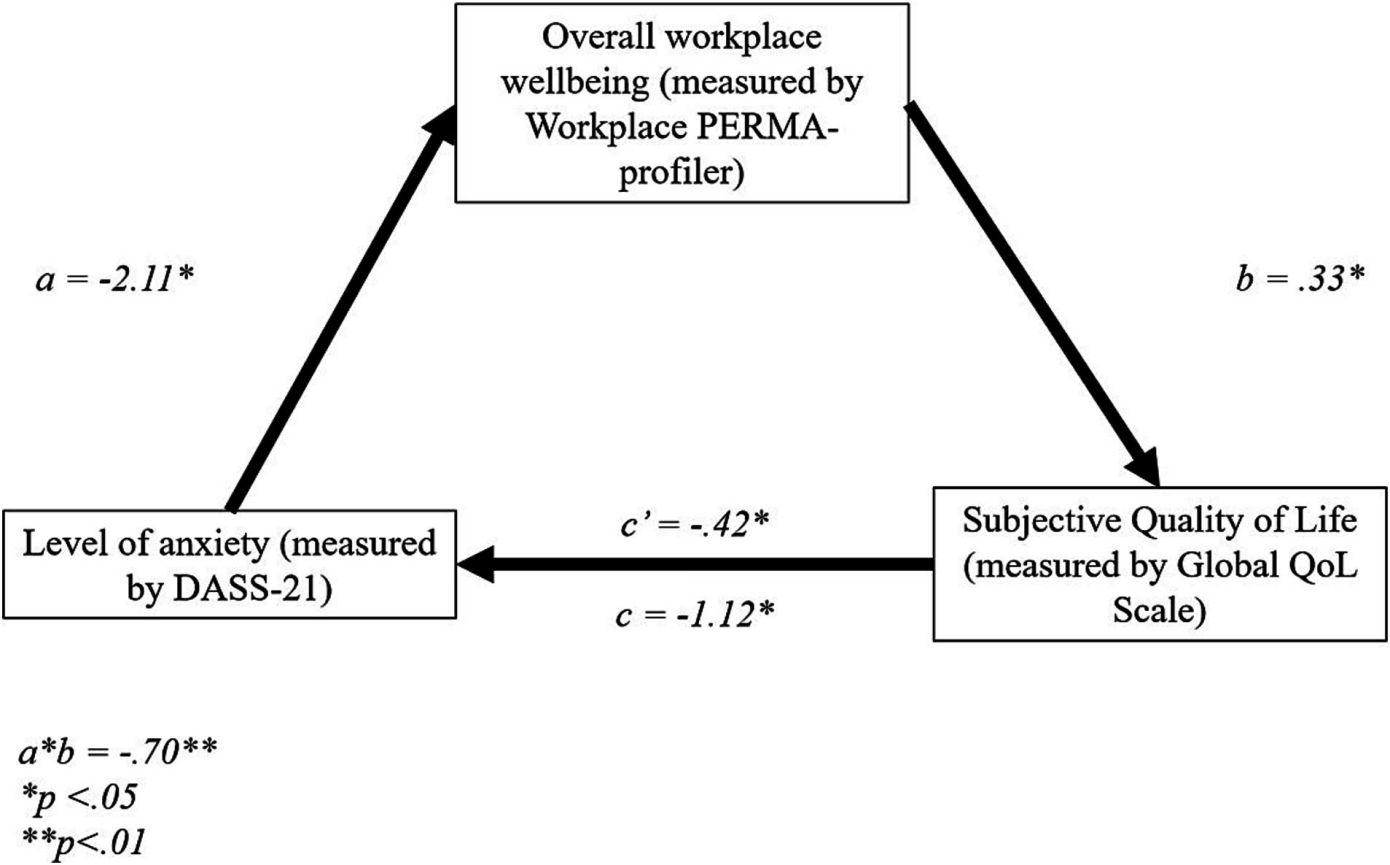
Standardized regression coefficients for the relationship between anxiety and sQoL. *p < 0.05, **p < 0.01.

The total effect model was significant, *b* = −1.12, *t* = −3.12, *p* < 0.01, 95% CI = −1.83, − 0.41. Finally, the indirect effect, *b* = −0.70, tested using a bootstrapping procedure with 5,000 samples (95% CI = −1.11, −0.37), was statistically significant (Figure 2). These results suggest that PERMA fully mediates the relationship between anxiety level and sQoL, thus supporting H_3_.

#### Mediating effect of workplace wellbeing on the relationship between stress levels and sQoL

There was a significant direct effect of stress level, as measured with the DASS-21, on overall workplace wellbeing, *b* = −2.85, *t* = −6.22, *p* < 0.01 (95% CI = −3.75, −1.95). There were further direct effects of overall workplace wellbeing on sQoL, *b* = 0.27, *t* = 0.04, *p* < 0.01, (95% CI = 0.19, 0.35), and stress level on sQoL, *b* = −1.27, *t* = −4.36, *p* < 0.01, (95% CI = −1.84, −0.69).

**Figure 3:**
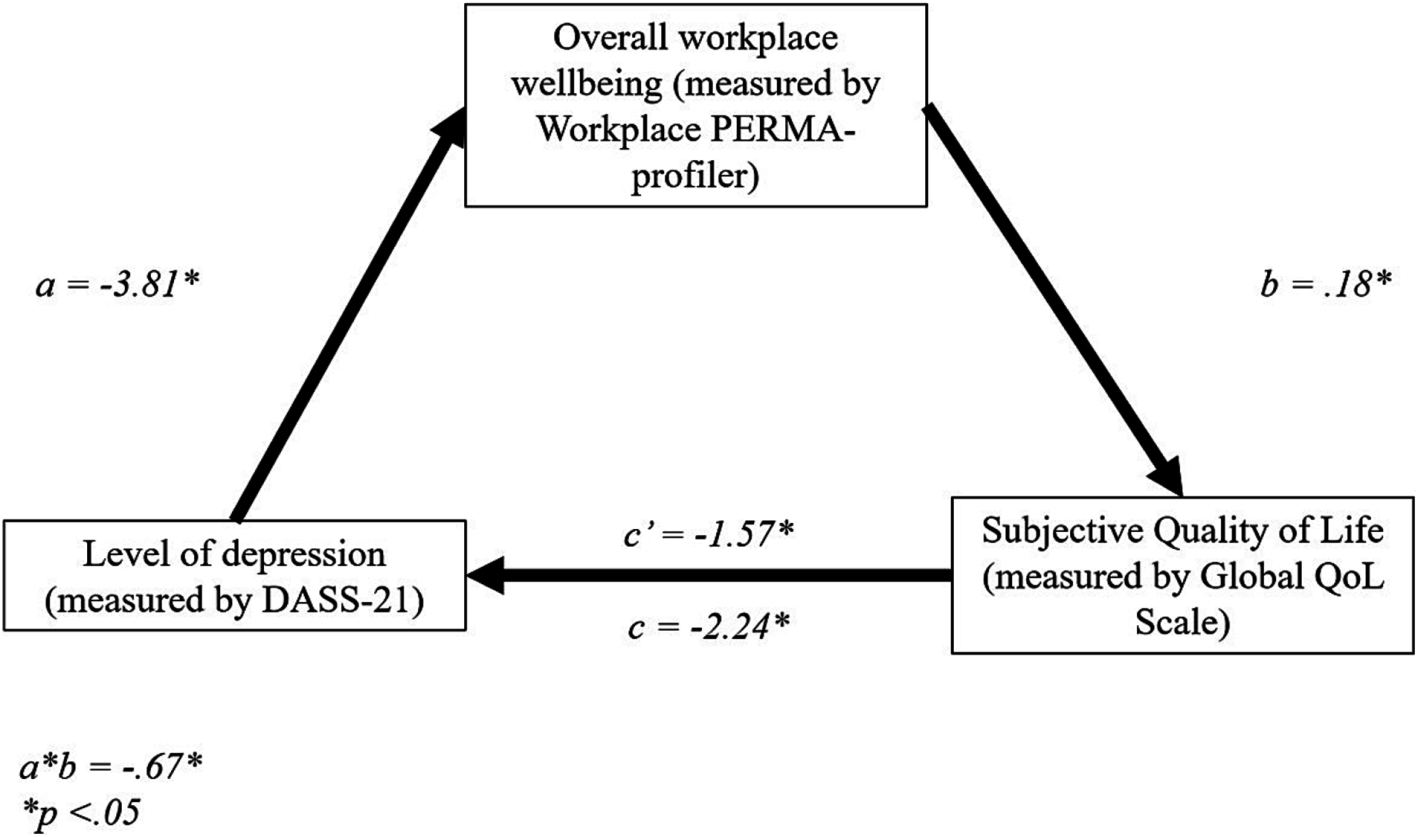
Standardized regression coefficients for the relationship between stress and sQoL. *p < 0.05, **p < 0.01.

The total effect model was significant, *b* = −2.04, *t* = −6.98, *p* < 0.01, 95% CI = −2.62, −1.46. Finally, the indirect effect, *b* = −0.77, tested using a bootstrapping procedure with 5,000 samples (95% CI = −1.23, −0.43), was statistically significant (Figure 3). These results suggest that PERMA partially mediates the relationship between stress level and sQoL, thus supporting H_3_.

### Qualitative analysis

This study aimed to explore how individuals within a diverse workplace conceptualize wellbeing. As such, participants were asked to define what wellbeing meant to them. Ritchie and Spencer's (1994) Framework Method, which integrates both inductive and deductive approaches to thematic analysis, was employed to develop themes from participants’ subjective perceptions of what wellbeing means and to determine whether new ideas relating to wellbeing would emerge beyond the PERMA framework. Initial code generation by two researchers yielded 49 individual codes. This was subsequently refined into 10 themes, which, when re-coded, resulted in a 97% inter-rater reliability rate. The remaining data were arbitrated by the third researcher, resulting in 477 statements from 214 responses, which fell into 10 themes (Table 8).

**Table 8:** Themes identified from qualitative analysis.

Origin of theme	Theme	Definition
Deductively drawn from the PERMA framework	Positive emotion	The experiencing of emotions such as happiness, hope, gratitude, optimism, contentment, and a sense of peace
	Engagement	Living in the present moment and immersing oneself in the task at hand; having and engaging in interests at or beyond work
	Relationships	Having authentic, meaningful, and loving relationships with other individuals
	Meaning	Having a sense of purpose and sense-making beyond the self (not necessarily in a religious or spiritual sense)
	Achievement	Experiencing a sense of accomplishment and satisfaction from the tasks that one chooses/engages in
Inductively drawn from open coding of responses	Psychological health	Being in good psychological health, the absence of psychological health concerns, and being able to cope with life stressors
	Physical health	Being in good physical health, the absence of disease, and being able to maintain one’s state of health
	Balance	Having the opportunity to navigate between both work and non-work activities and/or experiencing balance in multiple components of one’s life
	Meeting of basic needs	Availability of basic needs such as food, water, shelter, job stability, and other resources
	Autonomy	Being able to be independent of others and experiencing life without social pressure from others

The themes are presented below in terms of frequency of appearance, with Psychological Health (*N* = 104), Physical Health (*N* = 77), and Positive Emotion (*N* = 77) being the top three most common responses.

#### Psychological health

Psychological Health was the most frequently cited theme (*N* = 107). This theme consists of three distinct components, namely:

1. Being in good psychological health (example response: “(having a) healthy mental state).
2. The absence of poor psychological health (example response: “not always feeling anxious or stressed or in a fight or flight mood”).
3. Being able to cope with stressors and life demands (example response: “able to cope with work demands and stress”).

#### Physical health

Physical Health and Positive Emotion were in joint second position in terms of frequency in which the themes emerged (*N* = 77). Physical Health has three broad components, namely:

1. Being in a positive physical state (example response: “(having) high energy”).
2. Being free from physical ailments (example response: “free from sickness”).
3. Having the ability to maintain an optimal state of physical health (example response: “able to rest sufficiently and have a healthy diet”).

Notably, the themes of psychological health and physical health often converged, with many respondents mentioning them simultaneously. Example responses include:

*“Being mentally, emotionally, and physically balanced” and “Having a decent quality of life, both physically and mentally.”*

#### Positive emotion

Positive emotion was coded distinctly from Psychological Health, as it focused on the experiencing of desirable emotions and mood states, such as optimism and gratitude, rather than being in an optimal psychological state, the absence of psychological health concerns, or being able to cope with stressors. Example responses include “feeling generally comfortable or at peace with oneself” and “at peace with oneself and the world; get to experience positive emotions like happiness and excitement from time to time.”

#### Balance

A total of 47 respondents cited having balance as instrumental in helping them experience a sense of wellbeing. Balance consisted of two key components:

1. Being able to achieve equilibrium between work and life (example response: Balance in other components of existence beyond work and personal life).
2. Maintaining balance across multiple life domains beyond just work and personal life (example response: “Having time for rest, being able to spend quality time with loved ones, engaging in meaningful activities, feeling peaceful and having an overall balanced outlook and approach on life”).

#### Relationships

A total of 39 respondents noted that Relationships contributed to their sense of wellbeing. These respondents noted that they derived wellbeing from having meaningful and authentic relationships with others. Example responses include “(having) support from friends and family and an employer who listens” and “Being able to spend quality time with loved ones.”

#### Meeting of basic needs

Having basic needs attended to was indicated as a core component of wellbeing for 26 respondents. This entailed having access to food, shelter, jobs, and other resources. Example responses include “to comfortably support the lifestyle that I want for myself and my family financially,” “safe and secure housing, finances, community, employment,” and “having three square meals a day, four if I’m hungry, a bathroom, and a working internet connection.”

#### Autonomy

A total of 25 respondents noted that having a sense of Autonomy contributed to their sense of wellbeing. In this study, autonomy referred to the freedom to make independent choices without external pressure or imposed values. Example responses include “being able to live according to your personal needs and wishes rather than constraints imposed on you by your environment,” “good locus of control,” and “some extent of freedom of expression and not being tied down by anything.”

#### Engagement

A total of 21 respondents indicated that Engagement contributed to their wellbeing and highlighted the importance of being able to immerse oneself in one’s chosen activities. Example responses include “being able to work with the flow” and “being fully engaged at work.”

#### Meaning

A total of 19 respondents indicated that having a sense of meaning, or a sense of purpose and sense-making beyond the self, contributed to their sense of wellbeing. Example responses include “having a meaningful purpose,” “engaging in meaningful activities such as learning and playing,” and “spiritually, he/she is balanced with a good religious belief.”

#### Achievement

Finally, seven respondents indicated that Achievement, or experiencing a sense of accomplishment in one’s chosen tasks, was important for feeling a sense of wellbeing. Example responses include “thriving at work and in my personal life, achieving goals” and “a certain level of sense of achievement at work.”

## Discussion

This study demonstrated that individuals who suspected they had psychological health concerns (SPHC) reported lower workplace wellbeing and subjective quality of life (sQoL) than individuals who did not suspect having PHCs (nSPHC). Individuals who preferred not to say whether they suspected they had PHCs (SPNTS) also reported lower sQoL as compared to individuals who did not suspect they had a PHC (nSPHC). More importantly, the relationships between distress, as measured by the DASS-21, and sQoL were mediated by workplace wellbeing. Specifically, while the relationship between anxiety and sQoL was fully mediated by workplace wellbeing, the relationships between depression and sQoL and stress and sQoL were partially mediated.

Finally, the qualitative analysis indicated that Psychological Health, Physical Health, and Positive Emotion were the top three commonly cited contributors to wellbeing. Further contributors to wellbeing include Balance, Relationships, Meeting basic needs, Autonomy, Engagement, Meaning, and Achievement.

### Importance of a multifaceted conceptualization of wellbeing

The present research noted higher overall PERMA scores and individual PERMA facet scores in individuals from the nSPHC group compared to their counterparts from the SPHC group, with no single area being deemed less significant. This has implications on multiple levels. At the individual level, for those who experience SPHC but have yet to be diagnosed, conducting brief assessments and identifying gaps using the PERMA framework can also serve as a means of identifying areas of need and providing customized care. Interventions focusing on PERMA facets may then be helpful as a preventative strategy against psychological health concerns, with previous research showing the effectiveness of holistic PERMA-based interventions in mitigating psychological ill-health in areas such as distress, depression, anxiety, insomnia, and fear of disease progression in liver cancer patients (Guo et al., 2025). Such research suggests that interventions encompassing PERMA elements augment the current predominant symptomatic health management framework by emphasizing psychosocial elements beyond the mere absence of disease, thereby contributing to a more holistic healthcare model. On a broader level, within an organization, these results suggest that integrating PERMA elements and organizational policy may hold value in improving public health and wellbeing at a population level, such as through workplace wellbeing programs and policies.

### Suspecting having a psychological health concern

While the current study did not explicitly compare levels of distress in individuals who suspected they had PHCs to individuals who did not suspect having PHCs, the significantly lower PERMA and sQoL levels of SPHC as compared to nSPHC groups converge with previous literature that demonstrated that individuals who suspected that they had psychological health conditions had lower QoL and were more likely to meet clinically significant levels of psychological health symptoms as compared to individuals who did not suspect any psychological health conditions (Liu et al., 2020).

Individuals in the SPHC group experience lower sQoL, suggesting there is a potential impact of their psychological health state on their sQoL. Furthermore, while they have insight and are concerned about their psychological health, they may not have sought a formal diagnosis. Barriers to help-seeking in culturally diverse populations include the cost of treatment, the belief that treatment was unnecessary as the health concern would eventually subside, and fear of discrimination (Subramaniam et al., 2019). In addition to conducting further research into the barriers to help-seeking, interventions such as psychoeducation to increase mental health literacy, destigmatization, and motivational enhancement can be implemented to increase formal help-seeking behavior (Xu et al., 2018). Improving access to affordable psychological healthcare in the workplace and at a societal level is also warranted.

Currently, many companies offer subsidized rates for physical health checkups for their staff. As individuals become more aware of the importance of psychological health, it may also be beneficial to incorporate psychological health screening into employee benefit packages, allowing interested individuals to seek help if needed.

### Mediating effect of wellbeing on the relationship between depression, anxiety, and stress levels and sQoL

Previous literature has discussed how general wellbeing is specifically associated with work-related quality of life (QoL) (McFadden et al., 2021), while the current findings contribute to the research on how workplace PERMA mediates the relationship between depression, anxiety, stress levels, and subjective quality of life (sQoL). This adds to the literature that demonstrated PERMA mediates the relationship between depression and QoL (Choi, 2020). The present results provide implications on how potentially increasing workplace wellbeing can help to improve overall sQoL. This would benefit not only individuals but also organizations and the wider community, given that psychological health concerns and related losses in productivity are estimated to cost the global economy USD 2.5 trillion per year (The Lancet Global Health, 2020).

Regarding the differences between depression, anxiety, and stress, while workplace wellbeing only partially mediated the relationship between depression and stress and sQoL, it fully mediated the relationship between anxiety and sQoL. This suggests that workplace wellbeing interventions may be crucial in improving the sQoL of individuals with higher anxiety levels as compared to higher depression and stress levels. Interventions targeted at individuals with higher levels of anxiety may be particularly effective in increasing job productivity, especially given the higher level of anxiety as compared to depression in the workplace (Deady et al., 2021) and how anxiety is associated with increased levels of poorer work functioning and absenteeism (Plaisier et al., 2010). Nevertheless, it remains important to work toward workplace wellbeing for workers in general, as there are high comorbidity rates between anxiety and depression (Groen et al., 2020).

Notably, a study by Weziak-Bialowolska et al. (2020) suggests that there are bidirectional effects between life satisfaction and work satisfaction, with the former having a greater impact on the latter than vice versa. Given the impact of workplace wellbeing on sQoL in the present research, it may be helpful for future studies to consider the combined effect of both non-work and workplace wellbeing on QoL, especially in relation to the amount of time individuals spend in their work life. This may then help inform how wellbeing interventions can be applied in both individual and workplace settings.

### The function of non-disclosure

The rates of nondisclosure of suspected psychological health concerns (SPNTS = 10.1%, *N* = 20) in the present study appears on the higher end as compared to the rate of nondisclosure of other sensitive information, such as sexual activity and household income, ranging from 0.87 to 8.15% in past research (Tourangeau and Yan, 2007).

Respondents may have been cautious about divulging their psychological health status (PHS) for two reasons: first, it may be perceived as a sensitive topic, and second, respondents are concerned that the information they share may be disclosed to third parties (Tourangeau and Yan, 2007).

There were no differences in the sQoL of SPHC individuals and SPNTS individuals. However, both SPNTS and SPHC groups demonstrated significantly lower sQoL as compared to individuals who did not suspect they had psychological health conditions (nSPHC). This suggests that the SPNTS and SPHC groups are more similar to each other compared to the nSPHC group. While there are few studies on non-disclosure of PHS and levels of distress, previous research has demonstrated that non-disclosure of suicide ideation was associated with increased loneliness, poorer health, increased psychological distress, and more frequent suicide ideation (Mérelle et al., 2018). Importantly, Brouwers et al. (2019) suggests that employers can support employees with SPHCs but PNTS in the disclosure process by providing information about how they can disclose their PHC and who they should disclose it to so that individuals with PNTS may seek treatment more easily. Individuals who feel supported and are receiving treatment are also more likely to disclose PHS in the workplace (Reavley et al., 2017). It may prove more beneficial in workplace settings to facilitate ease of access to treatment (e.g., providing subsidized, anonymous therapy services) and foster healthy workplace relationships so that individuals who are hesitant to disclose their PHS would feel more comfortable seeking treatment and receive the support they need.

### Qualitative findings

Respondents in the current study were not primed to Seligman’s WBT. While the five PERMA facets did appear as coded responses, five additional themes were identified: Psychological Health, Physical Health, Balance, Meeting of Basic Needs, and Autonomy. These themes may be referred to as lay conceptualizations of wellbeing, or intuitive structures and theories by lay people to define and experience wellbeing, definitions which may differ from how theorists attempt to interpret wellbeing (McMahan et al., 2012). Interestingly, several lay theories proposed by participants in the present study align with additional domains proposed by Seligman’s WBT or the more recent PERMA+4 (Donaldson et al., 2022). These four additional domains are namely:

1. Physical health: Having positive physical health assets such as good genes and physical functioning.
2. Mindset: Having a growth mindset and the ability to overcome setbacks.
3. Work environment: A physical work environment that is suited to the individual’s needs that includes spatiotemporal elements such as sufficient space and natural light and.
4. Economic security: Experiencing financial stability and security in line with the individual’s needs (Donaldson et al., 2022).

The most frequently cited lay theory of wellbeing in the current study, *Psychological Health*, emphasizes the ability to cope with life stressors as a key component and bears resemblance to the PERMA+4’s proposed addition of *Mindset*. Psychological Health has previously been cited as a key lay conceptualization of wellbeing (Jarden et al., 2018), and such research, together with the current study, provides support for the proposed addition of this component as a PERMA+4 domain. Future studies may consider further exploring and refining the concept of psychological health and mindset for incorporation into the PERMA.

*Physical Health* in the present study was defined as being in good physical health, being free from physical ailments, and having the ability to maintain an optimal state of physical functioning. It is inherently similar to the definition of the PERMA+4’s conceptualization of *Physical Health*. The present study, along with others that also explore lay conceptualizations of wellbeing (Hone et al., 2015; Huang et al., 2022), provides support for incorporating physical health as a PERMA+4 domain.

*Economic Security* in the PERMA+4 also bears a resemblance to the current study’s theme of *Meeting Basic Needs*. It is supported by other studies on lay theories of wellbeing, citing the importance of concepts such as financial security (Downey, 2006) and infrastructural and food availability (Joshanloo et al., 2021) to human beings. Given the importance of cultural context in the development of lay theories (Joshanloo et al., 2021), the emergence of this theme may potentially indicate the current state of the world post-COVID, where job insecurity and financial anxiety are on the rise, justifying *Economic Security* as an additional facet of PERMA+4.

Apart from the similarities to the PERMA+4’s additional domains, the current study also identified other lay theories of wellbeing worthy of further exploration. Importantly, Positive Emotion and Psychological Health were coded as distinct themes, with Positive Emotion referring more to the experiencing of emotions such as happiness and peace, and Psychological Health referring to not just being in good psychological health but also being able to cope with adversity. The ability to cope with adverse experiences, at least in the present study, appears more frequently than the need to experience Positive Emotions. It is telling of the cultural diversity of the current population that these concepts appear as the two most important lay conceptualizations of wellbeing, given how Western-centric cultures tend to favor hedonistic notions of happiness (i.e., experiencing positive emotions) whilst many other cultures view suffering as an inevitable part of life (Joshanloo et al., 2021), possibly explaining here the necessity of being able to cope with challenges as a part of psychological health.

Balance was another frequently cited lay theory, referring not only to navigating between work and non-work activities but also to achieving balance in other areas outside of work and life (e.g., spiritual balance). While work-life balance has been defined as a lay theory in previous research (Hone et al., 2015), deeper notions of balance in non-Western cultures highlight the importance of maintaining a state of equilibrium between both positive and negative aspects while avoiding either extreme (Wong and Liu, 2018). Supporting this point, only one response in the data set defined wellbeing as not needing to work.

Autonomy was the final conceptualization of wellbeing, as derived from participants’ responses. While not part of Seligman’s WBT, autonomy appears as a key part of other wellbeing theories such as Ryff’s (1989) Scales of Psychological Wellbeing, which defines *Autonomy* as being self-determining and independent, and COMPAS-W, which defines *Own-Worth* as knowing one’s values and establishing healthy boundaries (Gatt et al., 2014). Autonomy may be a worthy contender for inclusion in the PERMA model in the future.

All in all, the above-derived lay theories of wellbeing demonstrate that, while there are parallels between general perception and theory, laypeople’s understanding of wellbeing adds a unique cultural and situational lens. It would then be important to carefully consider the distinct population in which wellbeing is being measured and craft interventions based on both theory and lay perceptions to cater to the identified population’s unique wellbeing needs.

At the same time, it may be important for researchers to acknowledge that there may never be a PERMA-nent understanding of wellbeing. Rather, wellbeing appears to be a constantly evolving concept, contingent upon time and space. Re-looking at definitions of wellbeing and refining wellbeing interventions may then need to occur periodically, especially in times of global upheaval, such as the COVID-19 pandemic.

### Study limitations and future directions

Regarding this study’s limitations, as the research focused on individuals who experienced SPHC, the experiences of individuals with diagnosed PHCs were not explored. There was also no comparison made between how wellbeing is defined by individuals with SPHC and individuals without SPHC. This is a pity, as Connell et al. (2014) suggest that wellbeing may be defined differently for people with and without diagnosed PHC. Future studies may wish to expand on comparisons between individuals with diagnosed PHCs, individuals experiencing SPHCs, and individuals who do not have or suspect having PHCs.

A second limitation in the present research is that demographic factors, such as age, ethnicity, and gender, and how they may manifest differently within the PERMA framework, were not explored. Few studies to date have thoroughly explored these differences, or if explored, findings have been inconsistent. For example, Burke and Minton (2019) observed that Irish male students had higher PERMA scores as compared to their female counterparts. However, Al-Hendawi et al. (2024) note negligible gender differences in PERMA scores. Exploring sociodemographic differences in wellbeing and quality of life, as well as the possible mechanisms behind these observable differences, is worthy of consideration in future research. In addition, research on what specific groups consider lay theories of wellbeing would also be valuable, as the present research has reflected that individuals’ definitions of wellbeing expand beyond current theoretical definitions. Such information would be invaluable in crafting suitable positive psychology interventions for specific groups, which, in turn, could benefit society as a whole.

Third, a missed opportunity in the present study was to further explore which factors might cause individuals to disclose PHC factors that hinder seeking help or diagnosis if they experienced SPHC, and how these areas relate to wellbeing. Doing so can help address issues related to help-seeking and interventions for individuals who experience SPHC.

Finally, future studies may wish to expand on which aspects contribute to a greater sense of wellbeing in various parts of the population (SPHC, no SPHC, SPNTS, diagnosed PHC). This is essential for developing interventions that promote both symptomatic and personal recovery.

## Data Availability

The original contributions presented in the study are included in the article/supplementary material, further inquiries can be directed to the corresponding author.
